# The use of acetabular screws in total hip arthroplasty and its influence on wear and periacetabular osteolysis in the long-term follow-up

**DOI:** 10.1007/s00264-021-05219-7

**Published:** 2021-09-28

**Authors:** Steffen Brodt, Kathleen Bischoff, Marcel Schulze, Dimitri Nowack, Andreas Roth, Georg Matziolis

**Affiliations:** 1 Department of Orthopedics, Jena University Hospital, Campus Eisenberg, Klosterlausnitzer Str. 81, 07607 Eisenberg, Germany; 2 Department Endoprosthesis/Orthopedics, Clinic of Orthopedics, Traumatology and Plastic Surgery, University Hospital Leipzig, Liebigstr. 20, 04103 Leipzig, Germany

**Keywords:** Hip, THA, Wear, Osteolysis, Screws

## Abstract

**Purpose:**

The cementless implantation of hip replacement cups may be performed with and without the additional use of acetabular screws. If the surgeon uses screws or not depends on variable factors. In general, the use of screws is intended to increase the primary stability of the cup. Whether screws increase the initial stability of the cup construct, or even reduce it in part, is the subject of considerable debate in the literature. It is also unclear whether the additional screws lead to increased wear or increased periacetabular osteolysis over the long-term course.

**Methods:**

Two hundred eleven patients from a previous study with a minimum follow-up of 10.7 years were included. Of these, 68 patients with 82 total hip arthroplasties (THA) were given clinical and radiological follow-up examinations. Of these, 52 had been fitted without screws and 30 with screws. On the basis of radiographs, annual wear and osteolysis were quantified. The clinical results were recorded by means of VAS, HHS, and WOMAC scores.

**Results:**

Significantly more periacetabular osteolysis was found if additive acetabular screws had been used. No difference was found in relation to the volumetric wear per year. Likewise, no difference was found with regard to the clinical scores.

**Conclusions:**

The use of additive acetabular screws leads to increased osteolysis in the periacetabular bone stock. Insofar as the primary stability of the cementless cup construct allows it, no additional acetabular screws should be used.

**Supplementary Information:**

The online version contains supplementary material available at 10.1007/s00264-021-05219-7.

## Introduction

Despite the continued excellent results of cemented acetabular cups, in most of the industrialised countries, cementless cups are implanted in the majority of cases. Different principles can be distinguished in the case of cementless technology. In the press-fit technique during the initial phase of so-called primary stability, a sufficient press-fit of the implant is required so that it can undergo lasting osseous integration over the further course (secondary stability) [1–4]. The initial press-fit of the cup is achieved by clamping in the area of the cup’s equator in the bony acetabulum. Here, in relation to the reaming depending on the cup model, the cup has an excess of around 1–3 mm [5, 6]. The additional screw fixation of press-fit cups is often applied in situations in which a reduced primary stability is expected, e.g. in the case of osteoporotic bone or a dysplastic acetabulum. The use of additional screws when implanting cementless cups in primary hip arthroplasty continues to be the subject of considerable debate in the literature [7–13]. In their investigations in 2015, Tabata et al. showed that the additional use of screws increased the primary stability of the cup [10]. However, FE analyses revealed that the use of screws only had a locally limited effect on the stability of the cup. Therefore, the authors considered it to be of little help to place several screws closely together [14]. As early as 1995, Won et al. came to the conclusion that additive acetabular screws do not necessarily increase the primary stability of the cup, but may even decrease it in the areas of the cup on the opposite side of the screws [15]. An instrumented registry analysis showed a higher revision rate during the first six years when screws were used. But after six years, the revision late with screws was lower [16]. Further, it is possible that polyethylene wear particles caused by backside wear may penetrate through the screw holes into the retroacetabular area and lead to osteolysis and, over the further course, to loosening [10]. Therefore, the primary objective of the present study was to determine whether, over the long-term course, the additional use of acetabular screws leads to increased wear of the polyethylene liner, measured on the basis of decentring of the prosthetic head in the liner. The secondary objective was to examine whether the use of acetabular screws leads to increased periacetabular osteolysis.

## Materials and methods

The study was registered in the German Clinical Trials Register (Deutsches Register Klinischer Studien) under the number DRKS00017666. Before the start of investigations, approval of the Ethics Committee of our University Hospital was obtained (No. 4514–08/15). A retrospective analysis was performed. For this purpose, we drew on a patient collective that had also been analysed by Roth et al. in 2006 [9]. Of the 211 patients included at that time, it was possible to include 68 patients (82 hips) in the present study. Of the remaining patients, 40 had died, 33 could not or did not wish to participate in the follow-up, without giving reasons, and 70 could no longer be contacted.

All patients gave their informed consent to participate in this investigation. In all patients, a transgluteal approach according to Bauer was used [17]. The operations were performed by a total of four different surgeons. Among the patients followed up, there were only five men. In a total of 14 patients, both hips were treated surgically. All patients were treated with the Duraloc® Sector cup in combination with an Enduron™ liner, which is made of conventional polyethylene (DePuy Synthes, Warsaw, IN, USA) [18, 19]. The cup consists of titanium and has a rough osteoinductive surface and variably utilisable screw holes. The screw holes have not been obliterated by the surgeons. All cups were underreamed by 2 mm. Two groups were formed. Group 1 was treated with ≥ one screw (1–3 screws) in the index operation (*n* = 30). Group 2 did not receive any screws (*n* = 52). In group 1, 19 patients were treated with one screw, ten with two, and only one patient with three screws. Because of that small number of cases, patients were distributed in just two groups (with and without screws). Cup sizes ranged from 38 to 56 mm with no differences between the two groups (*p* = 0.379). At the time of surgery, the average age of the patients was 56.0 ± 8.7 years (group 1: 53.8 years ± 8.0 years; group 2: 57.3 ± 8.9 years *p* = 0.078). The follow-up period was 16.7 ± 1.7 years in group 1 and 15.8 ± 2.1 years in group 2 (*p* = 0.057). The minimum follow-up was 10.7 years. No difference between the two groups was found with regard to height (group 1: 161.23 ± 8.0 cm; group 2: 160.25 ± 5.78 cm; *p* = 0.522), weight (group 1: 70.03 ± 11.57 kg; group 2: 69.19 ± 8.85 kg; *p* = 0.713), or the body mass index (BMI) (group 1: 27.04 ± 4.75 kg/m^2^; group 2: 26.96 ± 3.29 kg/m^2^; *p* = 0.927). At the time of the follow-up examination, standard in-house radiographs (pelvic survey and lateral view) were performed in all patients. On the basis of these images, the cup position was determined in inclination and anteversion, and the volumetric wear was measured in the sense of the decentration of the femoral head, using the software PolyWare (Draftware Inc., North Webster, IN, USA). The software mediCAD (Hectec GmbH, Altdorf, Germany) was used to quantify the periacetabular osteolysis. For this purpose, a classification into the 3 zones according to DeLee and Charnley was performed [20]. In a pre-study, the interobserver agreement for quantification of osteolysis was calculated from three independent observers. Interobserver agreement of quantification of osteolysis was calculated using SPSS (IBM SPSS Statistics 25, New York, USA). In addition, the Harris Hip Score (HHS), the Western Ontario and McMaster Universities Osteoarthritis Index (WOMAC), and pain on a visual analogue scale (VAS) were recorded. All measurement values were compared after testing for normal distribution using the unpaired *t*-test at a significance level of 0.05 (IBM SPSS Statistics 25, New York, USA).

## Results

**Fig. 1:**
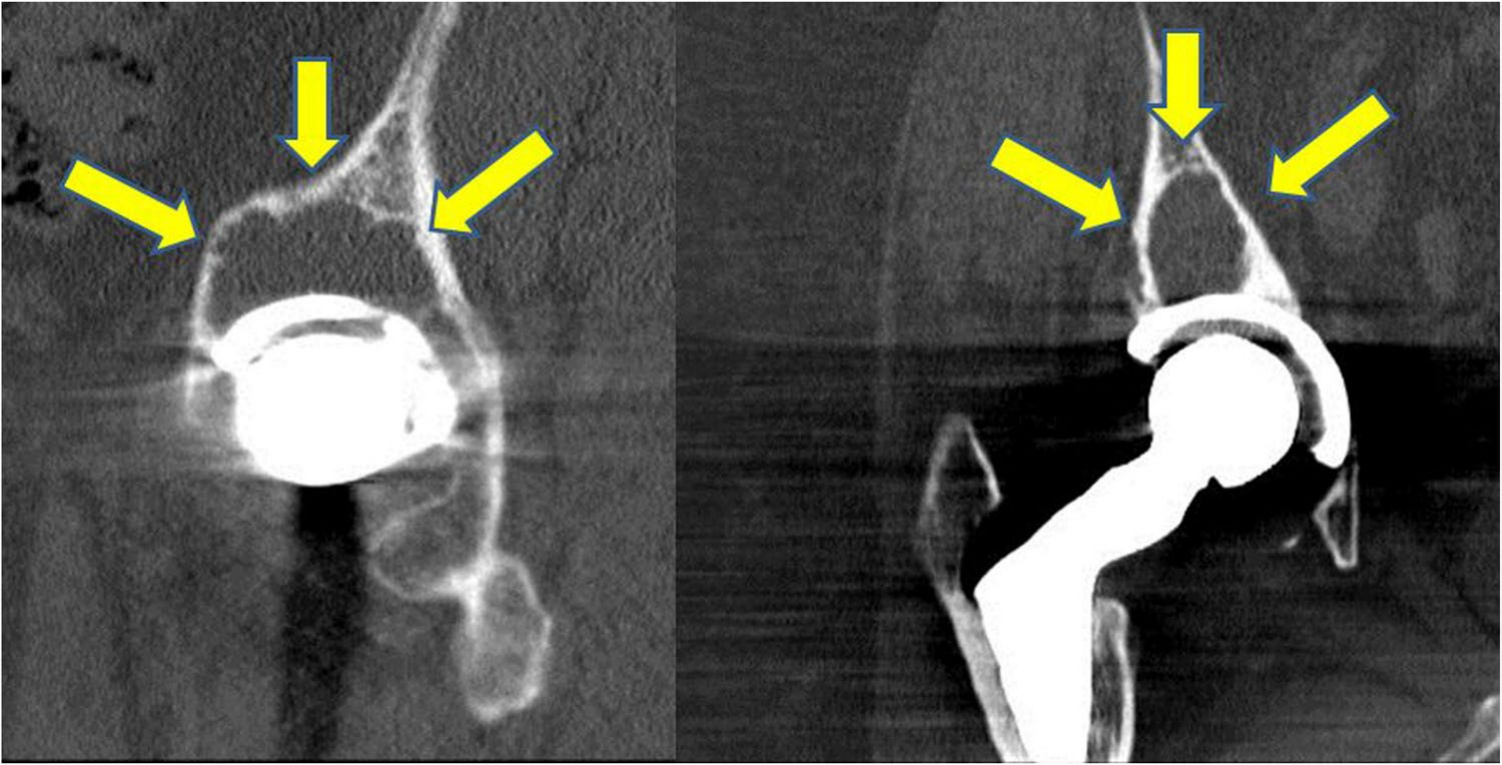
CT scan and sagittal and coronal reconstruction. Arrows indicating osteolysis

The interobserver agreement for quantification of osteolysis was calculated to be 0.827 for three observers (Cronbach’s alpha for metric variables). Alpha values between 0.8 and 0.9 represent a “good” internal consistency. No significant difference between the two groups was found with regard to the positioning of the acetabular cup. The average inclination measured with PolyWare was 45.2 ± 7.4° in group 1 and 43.8 ± 6.6° in group 2 (*p* = 0.373). The anteversion was 15.0 ± 8.3° in group 1 and 16.8 ± 8.1° (*p* = 0.333). The volumetric wear per year measured with PolyWare was 57.55 (range 4–222) ± 46.61 mm^3^/year in group 1 and 54.89 (range 0–166) ± 34.79 mm^3^/year in group 2 (*p* = 0.747). In contrast to this, there was a significant difference regarding the extent of periacetabular osteolysis. Here, in zone 2 according to DeLee and Charnley, osteolysis with an extent of 132.1 ± 80.8 mm^2^ was detected in group 1 compared to 87.1 ± 66.2 mm^2^ in group 2 (*p* = 0.007). Even considering the entire periacetabular bone from all zones, there were significant higher values found in group 1 (336.6 ± 133.7 mm^2^) in contrast to group 2 (266.0 ± 145.7 mm^2^) (*p* = 0.032). Group 1 showed that more than 25% of additional osteolysis of the surface were found than in group 2 (Table 1). During the follow-up period, a total of five inserts were changed due to wear and clinical complaints. Of these inserts, two were changed in group 1 and three were changed in group 2 (6.7% vs. 6.0%; *p* = 0.905 in the chi-square test). Operation reports of all revision cases were screened. In three reports, the surgeons described the finding of osteolysis. In one report, the size of the osteolysis was estimated at 2.5cm^3^. In this case, a CT scan showed the osteolysis pre-operatively (Fig. 1). At least in one of the two cases, where the surgeon did not describe osteolysis, the x-ray suggests the presence of osteolysis. In all cases, the cup was stable even in the presence of osteolysis. With regard to the PROM, no significant difference was found between the two groups (Table 2).

**Table 1:** Descriptive data, cup placement, wear, and osteolysis of group 1 (with screws) and group 2 (without screws)

	Group 1	Group 2
Number of patients	30	52
Age	53.8 ± 8.0 years	57.3 ± 8.9 years
Follow-up	16.7 ± 1.7 years	15.8 ± 2.1 years
Inclination	45.2 ± 7.4°	43.8 ± 6.6°
Anteversion	15.0 ± 8.3°	16.8 ± 8.1°
Wear rate	57.55 ± 46.61 mm^3^/year	54.89 ± 34.79 mm^3^/year
Osteolysis zone 1	132.1 ± 80.8mm^2^	87.1 ± 66.2mm^2^
Osteolysis total	336.6 ± 133.7mm^2^	266.0 ± 145.7 mm^2^

**Table 2:** Patient-related outcome measures of group 1 (with screws) and group 2 (without screws)

	Group 1	Group 2
Number of patients	30	52
NRS	1.85 ± 2.33	1.48 ± 1.88
HHS	77.0 ± 19.5	79.9 ± 14.4
WOMAC pain	1.32 ± 2.27	0.94 ± 1.40
WOMAC stiffness	2.08 ± 2.44	1.68 ± 2.01
WOMAC function	1.82 ± 2.45	1.76 ± 1.93
WOMAC global	1.74 ± 2.21	1.46 ± 1.64

## Discussion

Of utmost importance in cementless cup, implantation remains the achievement of high primary stability. Only if a cementless cup is seated in the bone stock without larger micromotion in the first weeks after implantation, reliable incorporation and thus the transition to secondary stability can be ensured. Numerous studies have shown that the additional use of acetabular screws can at least increase local stability initially [10, 14, 21]. The use of screws can lead to an extension and a prolongation of the operation. Additionally it still entails certain risks such as imprecise or unsuccessful screw placement with penetration of the bone and consecutively injury of nerves or vessels [22, 23]. However, attention must be paid to a precise placement of the screws here, as they may not only endanger the patient but may even reduce stability if they are not correctly placed. On the other hand, in the event of insufficient bone coverage, e.g. in a dysplastic or defect situation, screws may be helpful, just as in the case of poor bone quality. The cup used in this study has several screw holes that have not been obliterated by the surgeon intraoperatively when no screw was used. This can lead to facilitation of penetration of PE-wear particles into the retroacetabular bone. Osteolysis might be the consequence. To solve this problem, manufacturers nowadays offer cups with capped screw holes to minimise the risk of PE-particle penetration.

**Fig. 2:**
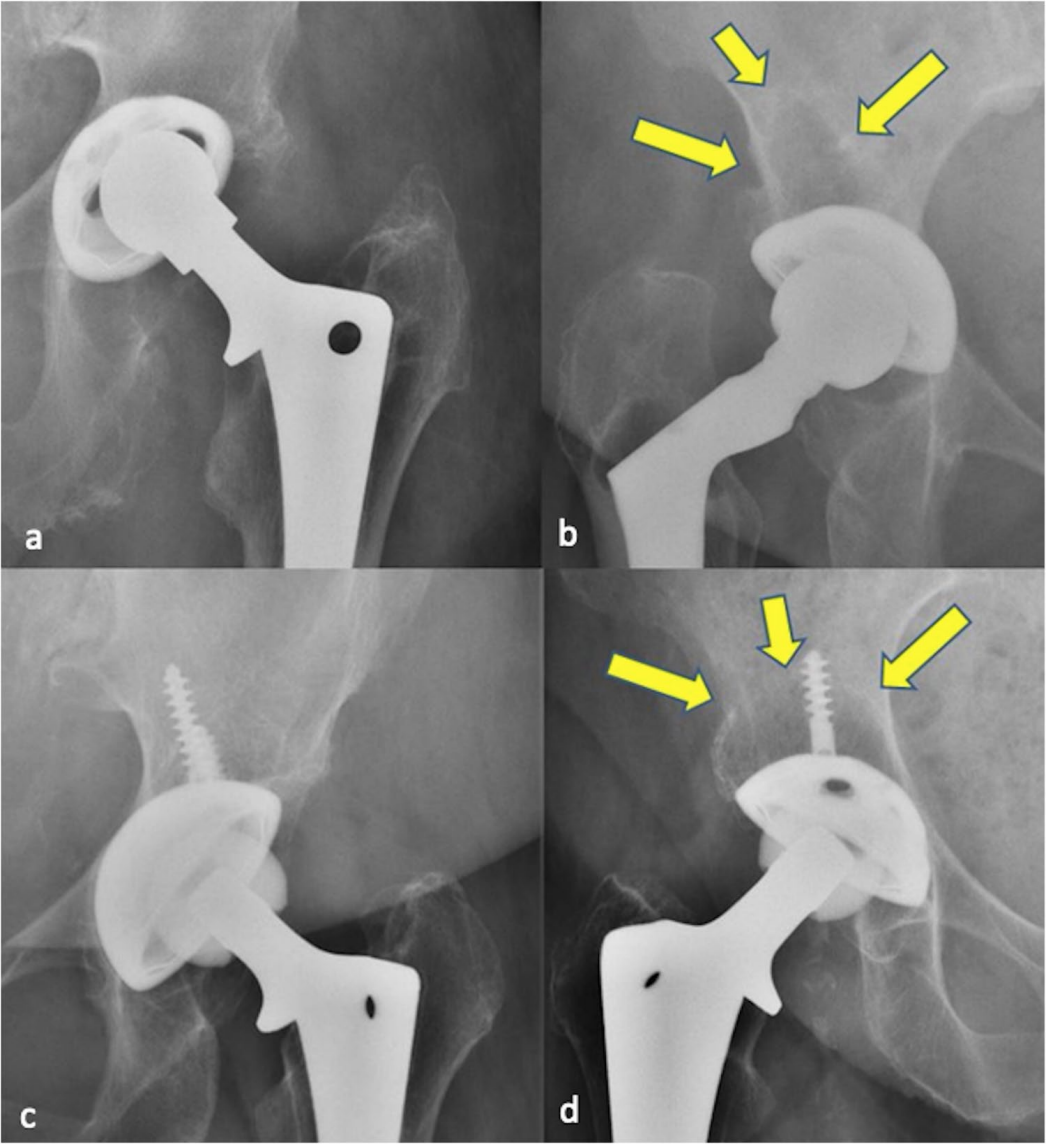
All possible combinations of screws and osteolysis were present in the patient collective: no osteolysis and no screw (**a**), osteolysis without screw (**b**), no osteolysis although screws (**c**), osteolysis in the presence of a screw (**d**)

The present study shows that the additional use of acetabular screws is not associated with a higher wear rate, but leads to significantly more osteolysis in the periacetabular bone stock in long-term follow-up. Figure 2 shows examples for osteolysis and no osteolysis with and without the presence of screws. In their study published in 2010, Iorio et al. also found more osteolysis when additive acetabular screws were used, even if the number of cases was small and significance could not be shown [24]. This result plays a role in particular over the long-term clinical course. Osteolysis increases the risk of aseptic loosening of the implant. If a revision operation then becomes necessary, the orthopaedic surgeon is confronted with reduced bone quality. However, good bone stock is absolutely essential for the reliable osseous anchorage of a revision implant. The surgeon can indeed take advantage of surgical techniques such as autologous bone grafting or augmentation with metallic implants. These possibilities are limited, and each further operation not only consumes more bone but also increases the peri-operative risks for the patient, such as infection, nerve lesions, or dislocation of the prosthesis. Therefore, every operation should be performed as sparingly and as minimally invasively as possible, and possible wear promoting factors should be considered and prevented.

This study has several limitations. As it is a retrospective study, confounders cannot be excluded. In particular, an inclusion bias might be present, leading to the bone quality at the time of implantation being poorer or a defect situation being present in the patient group treated with screws, which prevented a primary stability without screws. However, neither osteoporosis nor a bone defect is associated with periacetabular osteolysis regarding the literature, so that an influence on the study results is at least improbable. The long-term follow-up, which represents a strength of the study, is also responsible for the high number of patients who could not be re-examined (lost to follow-up). The high drop-out rate is a weakness of this investigation. Most of the elderly patient, we operated on, did not take part in the follow-up examination. That is why the average age of the patients seems to be quite young. The manual radiological evaluation is based on subjective criteria. The attempt was made to take this limitation into account by using two semi-automated programs (PolyWare and mediCAD). Moreover, a good interobserver agreement was determined for the quantification of osteolysis. The inserts examined in this study were made of conventional polyethylene. The wear rate detected in our study is comparable to the values found in other investigations [25, 26]. The authors are aware that the use of crosslinked PE has significantly reduced the wear rate and osteolysis in general, regardless of the use of screws [26–28]. Nevertheless, based on the results achieved in the present investigation, the authors recommend that, insofar as the primary stability of the implant allows it, the use of screws be done without.

## Supplementary Information

Below is the link to the electronic supplementary material.

Supplementary file1 (JPG 2000 KB)

## Data Availability

From the author.
