# Supplementary figures and images for: The use of acetabular screws in total hip arthroplasty and its influence on wear and periacetabular osteolysis in the long-term follow-up
Source: Int Orthop. 2021 Sep 28;46(4):717–22. doi: 10.1007/s00264-021-05219-7 (PMC8930858; doi:10.1007/s00264-021-05219-7)

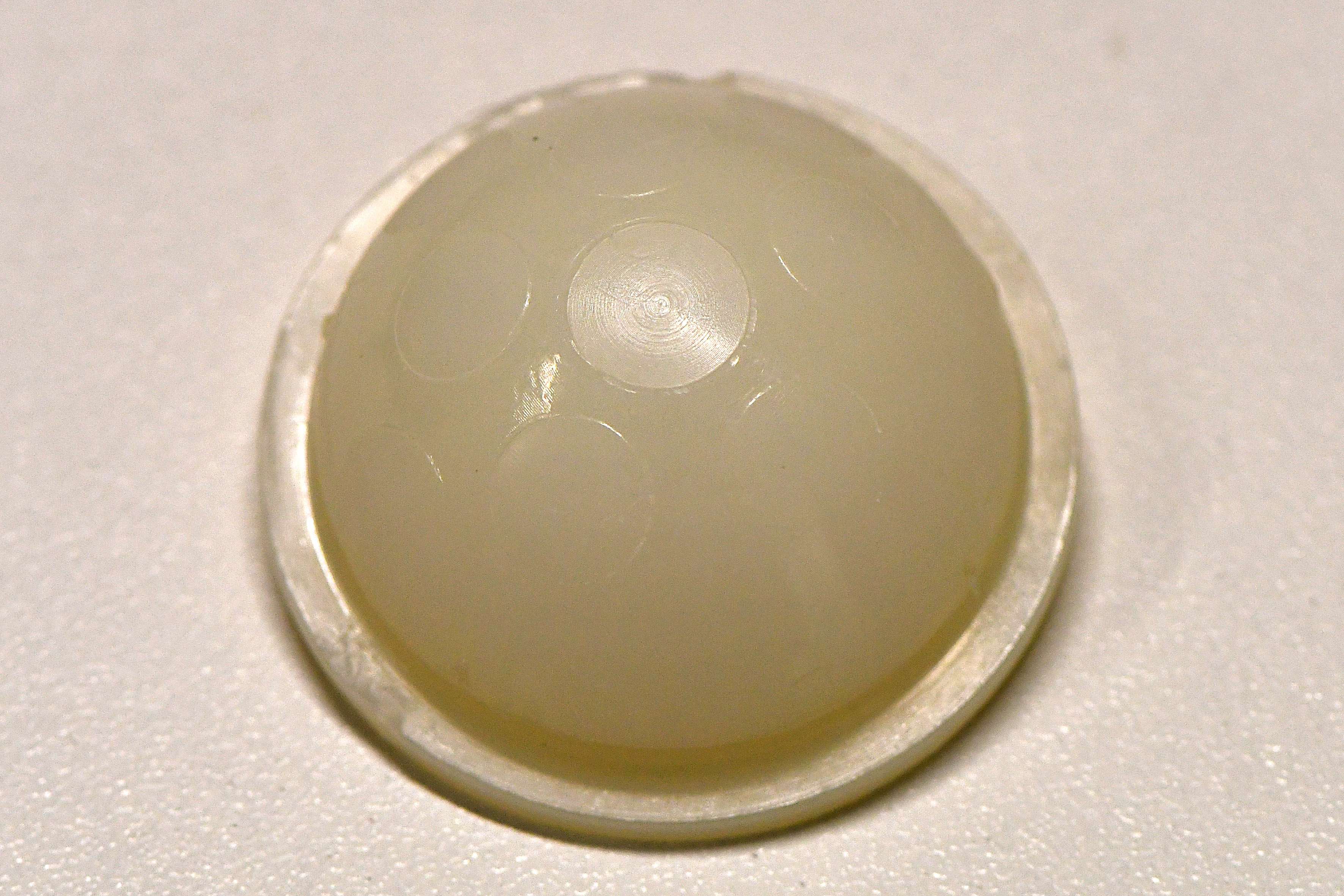

Supplement: Supplementary file 1 — Supplementary file1 (JPG 2000 KB) [file 264_2021_5219_MOESM1_ESM.jpg]
